# Rectifying misconceptions and misimplementations: A critical examination of health literacy interventions in health systems

**DOI:** 10.34172/hpp.025.45371

**Published:** 2025-11-04

**Authors:** Hamid Allahverdipour

**Affiliations:** ^1^Editor-in-Chief, Health Promotion Perspectives; ^2^Department of Health Education & Promotion, Tabriz University of Medical Sciences, Tabriz 14711, Iran

 The persistent burden of poor health outcomes attributable to limited health literacy underscores a critical failure in systemic intervention strategies. For decades, the prevailing approach within healthcare systems has been analogous to addressing symptoms while neglecting the underlying etiology. We have acknowledged the manifest struggles individuals face in navigating health information, yet we have fundamentally misattributed the primary cause, leading to insufficient and often ineffective solutions. While the growing emphasis on health literacy represents a necessary evolution in healthcare delivery, its potential is critically undermined by pervasive conceptual misconceptions and, more operationally, by profound implementation failures. To genuinely advance health equity and improve patient outcomes, a paradigm shift is required—one that repositions the problem from an individual deficit to a systemic characteristic.

 The most significant misconception obstructing progress is the framing of health literacy as a patient-centric deficit. This paradigm locates the problem within the individual’s skills and knowledge, implying a solution focused on “remediating” the patient through simplified information dissemination. However, contemporary scholarship defines health literacy not as a static individual trait, but as a dynamic product of the interaction between an individual’s capacities and the complexities and demands of the healthcare system.^1^ A system that necessitates advanced literacy skills to decipher insurance documentation or relies on dense medical jargon during high-stakes consultations is, by definition, a system demonstrating low health literacy. A singular focus on patient comprehension inadvertently absolves the system of its fundamental responsibility to be comprehensible.

 This foundational error in conceptualization precipitates a series of misimplementations—systemic failures in execution that render interventions ineffective. A predominant misimplementation is the propensity for isolated, “one-off” initiatives. An institution may invest substantially in developing elegantly designed, low-literacy educational materials for a specific condition, such as diabetes, while concurrently maintaining a convoluted appointment scheduling process, impenetrable hospital discharge protocols, and clinical communication styles reliant on inaccessible terminology. This siloed strategy is architecturally unsound; it is comparable to installing a single accessibility feature while ignoring the overall infrastructure of barriers that renders the environment navigable only to a privileged few.

 Further critical misimplementations are prevalent. Interventions are frequently developed without the foundational process of co-design with target communities, resulting in materials and protocols that are culturally incongruent or practically misaligned with lived realities.^2^ An over-reliance on written materials persists, often without complementary investment in training frontline staff in evidence-based interpersonal communication techniques, such as the Teach-Back method, which is a cornerstone of verifying patient understanding.^3^ Moreover, health literacy is often marginalized as an issue pertinent only to specific, frequently stigmatized subgroups, rather than being embraced as a universal precaution. Just as standard infection control procedures are applied to all patients, clear communication strategies must be universally employed, as the capacity to process complex health information under stress cannot be reliably discerned.^4^

 The digital transformation of healthcare introduces a further layer of complexity: the digital health literacy divide. The rapid proliferation of patient portals and telehealth services presupposes universal access, digital competence, and trust, thereby potentially exacerbating disparities and systematically excluding the very populations most vulnerable to the effects of low health literacy.^5^

 A fundamental reorientation is therefore imperative. The path forward necessitates a deliberate and systematic shift in strategy. First, a transition from fragmented projects to systemic integration is essential. Health literacy must be elevated from a peripheral initiative to a core organizational competency, embedded into the very fabric of health system operations—from high-level strategic planning and health information technology design to staff training protocols and physical wayfinding.

 Second, the adoption of participatory co-design methodologies is non-negotiable. Patients and community stakeholders must be reconceptualized from passive recipients of care to essential partners in the design and evaluation of interventions. Their lived experience constitutes invaluable data for identifying systemic failures and developing efficacious solutions.

 Third, the mandatory and continuous development of communication skills across the workforce is critical. Training in plain language, cultural humility, and structured communication techniques like the Teach-Back method must be standardized and sustained for all clinical and administrative personnel.

 In conclusion, the evidence is unequivocal: poor health literacy is a significant driver of escalating healthcare costs, suboptimal outcomes, and entrenched health inequities. The solution, however, extends beyond the production of refined patient education materials. The imperative is to architect more intelligible, humane, and responsive health systems. It is time to cease the futile endeavor of merely teaching patients to navigate an incomprehensible maze and begin the necessary work of dismantling and redesigning the maze itself.

## Competing Interests

 Hamid Allahverdipour is the Editor-in-Chief of Health Promotion Perspectives.

## Ethical Approval

 Not applicable.
